# Anchoring and Catalytic Performance of Co@C_2_N Monolayer for Rechargeable Li-Se*_x_*S*_y_* Batteries: A First-Principles Calculations

**DOI:** 10.3390/molecules29225264

**Published:** 2024-11-07

**Authors:** Xiaojing Li, Yingbo Zhang, Chenchen Liu, Shuwei Tang

**Affiliations:** 1 Department of Automotive Engineering, Hebei Petroleum University of Technology, Chengde 067000, China; lixiaojingre@163.com; 2 College of Materials Science and Engineering, Liaoning Technical University, Fuxin 123000, China; liuchenchen0801@163.com

**Keywords:** Li-Se*_x_*S*_y_* batteries, Co@C_2_N monolayer, shuttle effect, Li_2_Se*_x_*S*_y_*/Se*_x_*S*_y_*, first-principles calculations

## Abstract

Se*_x_*S*_y_* composite cathode materials, which offer superior theoretical capacity compared to pure selenium and improved electrochemical properties relative to pure sulfur, have aroused considerable interest in recent decades on account of their applications in electric vehicles and energy storage grids. In the current work, the feasibility of a Co@C_2_N monolayer as a promising host candidate for the cathode material of Li-Se*_x_*S*_y_* batteries has been evaluated using first-principles calculations, and particular efforts have been devoted to underscoring the anchoring mechanism and catalytic performance of the Co@C_2_N monolayer. The pronounced synergistic effects of Co-S and Li-N bonds lead to increased anchoring performance for Li_2_Se*_x_*S*_y_*/Se*_x_*S*_y_* clusters on the surface of Co@C_2_N monolayer, which effectively inhibit the shuttle effect. The charge density difference and Mulliken charge analysis underscores a substantial charge transfer from the Li_2_Se*_x_*S*_y_* and Se*_x_*S*_y_* clusters to the Co@C_2_N monolayer, which indicates a noticeable chemical interaction between them. Further electronic property calculations show that the Co@C_2_N monolayer can improve the electrical conductivity of cathode materials for Li-Se*_x_*S*_y_* batteries by maintaining semi-metallic characteristics after anchoring of Li_2_Se*_x_*S*_y_*/Se*_x_*S*_y_* clusters. Additionally, the catalytic performance of the Co@C_2_N monolayer is evaluated in terms of the reduction pathway of Se_8_ and the decomposition energy barrier of the Li_2_SeS cluster, which highlights the catalytic role of the Co@C_2_N monolayer in the formation and decomposition of the Li_2_SeS cluster during the cycle processes. Overall, the Co@C_2_N monolayer emerges as a promising host material and catalyst for Li-Se*_x_*S*_y_* batteries with remarkable anchoring and catalytic performance.

## 1. Introduction

Escalating global energy scarcity has spurred a concerted effort to innovate new energy technologies with the aim of addressing the energy crisis and satisfying the demand for eco-friendly energy solutions [1,2]. Lithium-ion batteries have emerged as vital players in fulfilling the surging needs of portable devices, electric vehicles, and energy storage grids, owing to their long cycle life, small size, high energy density, and minimal environmental pollution. However, the energy output provided by the renewable inserted lithium-ion batteries, even at peak development, falls short of increasing market demand [3,4,5,6,7]. Therefore, it is imperative to develop a new generation of cost-effective lithium-ion batteries to provide higher energy density. Notably, the chalcogens, such as oxygen, sulfur, and selenium, are abundant in nature and boast remarkable energy densities, which positions them as promising candidates for future energy storage applications. However, Li-O batteries encounter a series of challenges, including poor cycling performance, electrolyte decomposition, excessive lithium for anode, and large voltage polarization [8,9], which impede the widespread application of Li-O batteries. Remarkably, the rechargeable Li-S and Li-Se batteries emerge as the most promising contenders. Li-S batteries garner considerable attention not only because of their high specific capacity (1672 mAh·g^−1^) and energy density (2600 Wh·kg^−1^) [10] but also for their affordability, abundant reserves, low toxicity, and environmental friendliness. Nevertheless, with the further study of Li-S batteries, there are drawbacks to Li-S batteries, such as low conductivity of sulfur, the discharge intermediate products, the large volume change during the charging/discharging process, and the shuttle effect of high-order polysulfides, which diminish the sulfur utilization rate and Coulombic efficiency [11,12,13,14,15]. Selenium, akin to sulfur in chemical properties, emerges as a prospective cathode material for volume-sensitive applications. Importantly, Li-Se batteries exhibit superior electrical conductivity (1 × 10^−3^ S·m^−1^) and enhanced electrochemical activity compared to Li-S batteries (5 × 10^−28^ S·m^−1^) [16]. Furthermore, Li-Se batteries boast obvious advantages, such as heightened active material utilization rate, robust rate capacity [17], and favorable electrochemical compatibility with conventional liquid electrolytes [18,19,20].

Amidst the fervent exploration of cathode materials for Li-S and Li-Se batteries, a kind of novel rechargeable Li-Se*_x_*S*_y_* batteries has emerged. Se*_x_*S*_y_* cathode materials exhibit a superior theoretical capacity compared to their isolated selenium counterpart and enhanced conductivity compared to pure sulfur cathodes [21]. Moreover, Sun et al. [22] demonstrated the performance of cathode materials with varying Se/S ratios in diverse ether-based electrolytes, highlighting the significant enhancement of Se_2_S_6_ materials in cycling performance. Therefore, the Se*_x_*S*_y_* composite emerges as a promising cathode material with prolonged cycle life and heightened power density. Despite the notable improvement in the cycle stability facilitated by the Se*_x_*S*_y_* composite, the initial Coulombic efficiency of the Se*_x_*S*_y_*/C composite remains low, which may be attributed to the side-reactions of sulfide/selenide with the electrolytes. Moreover, the weak interaction between the Se*_x_*S*_y_* composite and the carbon host material leads to the ineffective confinement of selenium and sulfur materials. Therefore, developing a novel host material for anchoring polyselenide/polysulphide intermediates becomes a matter of great urgency in Li-Se*_x_*S*_y_* batteries.

To address the challenges associated with volume expansion and shuttle effect in Li-Se*_x_*S*_y_* batteries, a prevalent strategy, namely developing different forms of carbon electrodes by using nano-sized carbon materials [23,24], is adopted. Carbon graphitization not only helps to trap selenium and sulfur active materials but also facilitates electrochemical activity of carbon through *sp*^2^ hybridization. Moreover, a composite material formed by combining carbon with other atoms can also improve the electrochemical performance of Li-Se*_x_*S*_y_* batteries while accommodating their volume expansion during discharge. The typical example is the X (X = N, O, P) atoms doping carbon materials, which can not only suppress the shuttle effect but also enhance the electrical conductivity [25,26,27,28,29]. Very recently, a novel carbon nitride material, layered C_2_N material with vesicular configuration and high nitrogen concentration, has garnered considerable attention in the field of energy storage on account of its expansive surface area and excellent structural, electrical, and mechanical properties.

By first-principles calculations, the anchoring mechanism of polyselenides on a C_2_N monolayer [30] was systemically investigated, revealing a robust binding interaction between the C_2_N monolayer and polyselenides. Similarly, a subsequent work explored by Lin et al. [31] further evaluated the feasibility of a transition metal-doped C_2_N monolayer as a cathode material for Li-S batteries, demonstrating the highest adsorption energy and the lowest decomposition energy. Importantly, a recent theoretical work [32] substantiated that a Co@C_2_N monolayer can mitigate the shuttle effect of high-order polyselenides, accelerate Li_2_Se conversion, and improve the cycling performance of Li-Se batteries. Considering the similarity of Li-Se*_x_*S*_y_* batteries to Li-S or Li-Se batteries, the Co@C_2_N monolayer is also expected to be a promising cathode material. Nevertheless, the design and application of Co@C_2_N monolayers in Li-Se*_x_*S*_y_* batteries are still at a very early stage, and few works concerning on the anchoring and catalytic performance of Co@C_2_N monolayers have been reported so far. Therefore, in the current work, the feasibility of a Co@C_2_N monolayer as a cathode material for Li-Se*_x_*S*_y_* batteries is evaluated through first-principles calculations, and particular attention is paid to the exploration of the anchoring effect and catalytic performance. The binding energies, charge transfer mechanism, and electronic properties of Li_2_Se*_x_*S*_y_*/Se*_x_*S*_y_* clusters anchored on a Co@C_2_N monolayer are investigated. The present work could provide valuable insights into the potential development of Co@C_2_N monolayers as cathode materials for high-performance Li-Se*_x_*S*_y_* batteries.

## 2. Results and Discussion

### 2.1. Pristine C_2_N, Co@C_2_N Monolayer, and Li_2_Se_x_S_y_ (Se_x_S_y_) Cluster Structures

**Figure 1:**
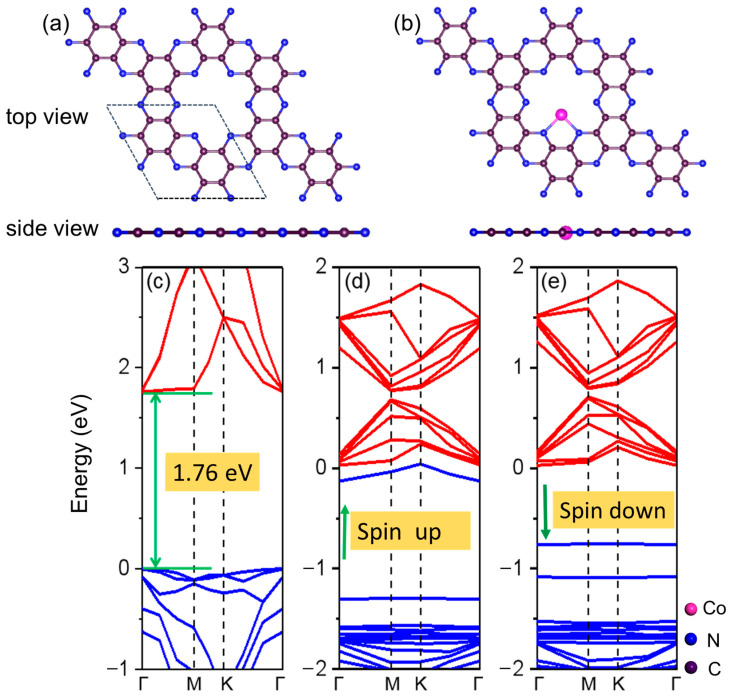
(**a**,**b**) The optimized structures of intrinsic C_2_N and Co@C_2_N monolayers. The magenta, blue, and purple circles denote the Co, N, and C atoms, respectively. (**c**–**e**) The electronic band structures of (**c**) intrinsic C_2_N and (**d**) spin-up and (**e**) spin-down states of Co@C_2_N monolayers along the high symmetrical Γ-Μ-Κ-Γ path. Reproduced with permission from the Journal “Nanoscale”/Royal Society of Chemistry, ref. [32].

Figure 1a depicts the optimized configuration of the C_2_N monolayer, which comprises 12 carbon and 6 nitrogen atoms, and the lattice parameters (*a* = *b* = 8.332 Å) are consistent with the theoretical and experimental values (8.300 Å) [32,33].

**Figure 2:**
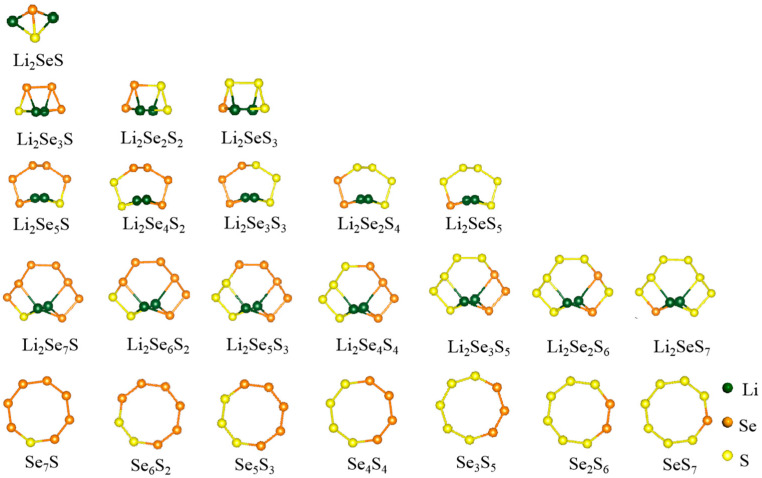
The most stable structures of Li_2_Se*_x_*S*_y_* and Se*_x_*S*_y_* (*x* + *y* = 2, 4, 6, 8) clusters.

For the favorable position of the Co atom within the C_2_N monolayer, the Co@C_2_N monolayer (Figure 1b) adopted in the previous work [34], where Co atom bonds with adjacent N atoms, is selected as the candidate cathode material of the Li-Se*_x_*S*_y_* batteries. Figure 1c–e illustrate the electronic band structures of a pristine C_2_N monolayer and the Co@C_2_N monolayer. Obviously, the pristine C_2_N monolayer manifests a direct band gap semiconductor with a band gap of 1.76 eV, and the embedded Co atom reduces the band gap of the C_2_N monolayer, imparting semi-metallic characteristics to the Co@C_2_N monolayer. During the discharge process, Li^+^ undergoes reactions with sulfur and selenium to form a series of selenium-containing and sulfur-containing compounds, as presented in Table S1 of the Supporting Information, and Figure 2 depicts the most stable configurations of Li_2_Se*_x_*S*_y_* and Se*_x_*S*_y_* (*x* + *y* = 2, 4, 6, 8) clusters, in which the sulfur atom prefers to bond with sulfur atoms and lithium atoms are inclined to bond with sulfur and selenium atoms.

### 2.2. Geometries, Binding Energies of Li_2_Se_x_S_y_ (Se_x_S_y_) on Co@C_2_N Monolayer

**Figure 3:**
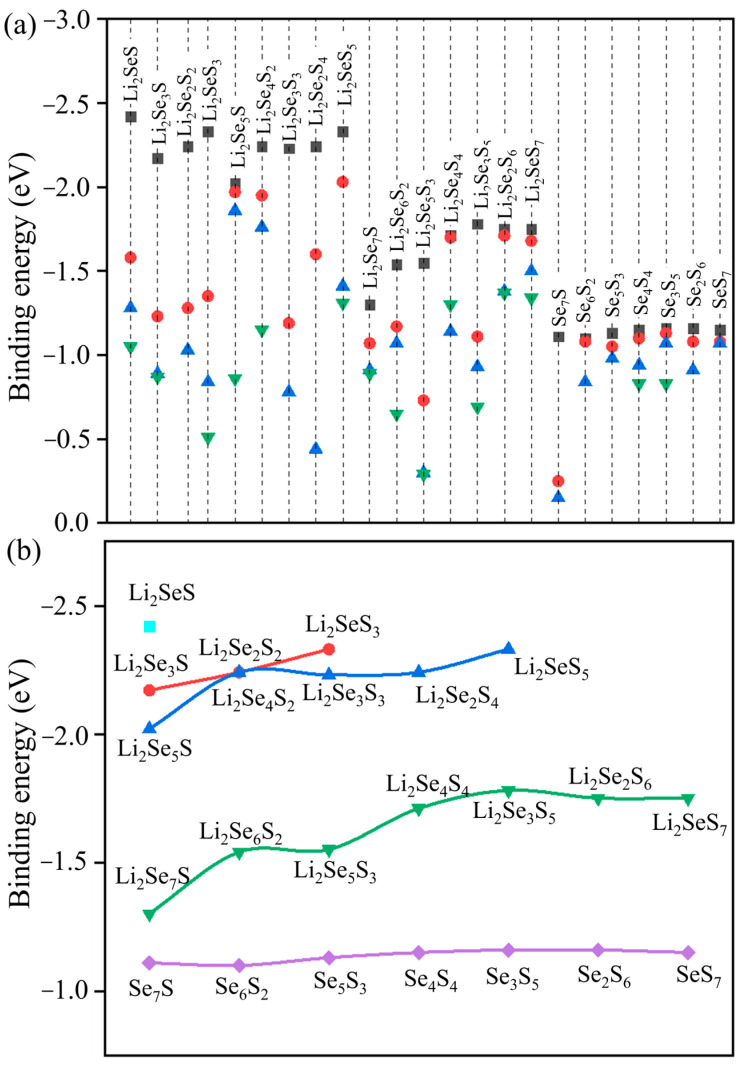
(**a**) Binding energies of Se*_x_*S*_y_* and Li_2_Se*_x_*S*_y_* (*x* + *y* = 2, 4, 6, 8) clusters anchored on the surface of the Co@C_2_N monolayer at different adsorption positions. (**b**) Binding energies of the most stable Se*_x_*S*_y_* and Li_2_Se*_x_*S*_y_* (*x* + *y* = 2, 4, 6, 8) clusters on the surface of the Co@C_2_N monolayer.

To investigate the feasibility of utilizing the Co@C_2_N monolayer as a cathode material for Li-Se*_x_*S*_y_* batteries, the most suitable adsorption sites for Li_2_Se*_x_*S*_y_* and Se*_x_*S*_y_* (*x* + *y* = 2, 4, 6, 8) clusters on Co@C_2_N monolayer are firstly explored by binding energies (*E*_b_) [35] using the following definition:

(1) $$E_{b}=E_{Co@C_{2}N+Li_{2}Se_{x}S_{y}/Se_{x}S_{y}}-(E_{Li_{2}Se_{x}S_{y}/Se_{x}S_{y}}+E_{Co@C_{2}N})$$

where the $E_{Li_{2}Se_{x}S_{y}/Se_{x}S_{y}}$, $E_{Co@C_{2}N}$, $E_{Co@C_{2}N+Li_{2}Se_{x}S_{y}/Se_{x}S_{y}}$ represent the total energies of Li_2_Se*_x_*S*_y_* or Se*_x_*S*_y_* clusters, the Co@C_2_N monolayer, and the total energies of Li_2_Se*_x_*S*_y_* and Se*_x_*S*_y_* molecules adsorbed on the Co@C_2_N monolayer, respectively. The *E*_b_s for the Li_2_Se*_x_*S*_y_* and Se*_x_*S*_y_* (*x* + *y* = 2, 4, 6, 8) clusters at different anchoring sites on the surface of the Co@C_2_N monolayer are depicted in Figure 3a, and the corresponding adsorbed configurations associated with binding energies are presented in Table S2 of the Supporting Information. Obviously, negative *E*_b_s are observed for Co@C_2_N-Li_2_Se*_x_*S*_y_* (Se*_x_*S*_y_*) (*x* + *y* = 2, 4, 6, 8), indicating the robust anchoring capability for Li_2_Se*_x_*S*_y_* and Se*_x_*S*_y_* clusters on the surface of the Co@C_2_N monolayer. More importantly, a positive correlation is observed for the *E*_b_ and the concentration of S atoms, i.e., an increase in the number of S atoms correlates with heightened *E*_b_ values for the Co@C_2_N-Li_2_Se*_x_*S*_y_*/Se*_x_*S*_y_*, indicating the enhanced anchoring effect. As the lithium reaction progresses from the Li_2_Se*_x_*S*_y_* (*x* + *y* = 8) to Li_2_SeS, an overall increase is observed for the *E*_b_s for most stable Co@C_2_N-Li_2_Se*_x_*S*_y_* (*x* + *y* = 2, 4, 6, 8) configurations, which underscores the robust and strong adsorption strength of the Co@C_2_N monolayer for Li_2_Se*_x_*S*_y_* clusters. Figure 3b illustrates corresponding *E*_b_s for the most stable configurations of Li_2_Se*_x_*S*_y_* and Se*_x_*S*_y_* clusters anchored on the Co@C_2_N monolayer, which span from −1.30 eV of Li_2_Se_7_S to −2.42 eV for Li_2_SeS cluster. Moreover, the *E*_b_s for Li_2_Se*_x_*S*_y_* clusters anchored on the Co@C_2_N monolayer increases with the lithiation and decreases with the reduction in the concentration of S atoms, suggesting the contributory role of S atoms in bolstering the binding strength between Li_2_Se*_x_*S*_y_* clusters and the Co@C_2_N monolayer and consequently suppressing the shuttle effect.

**Figure 4:**
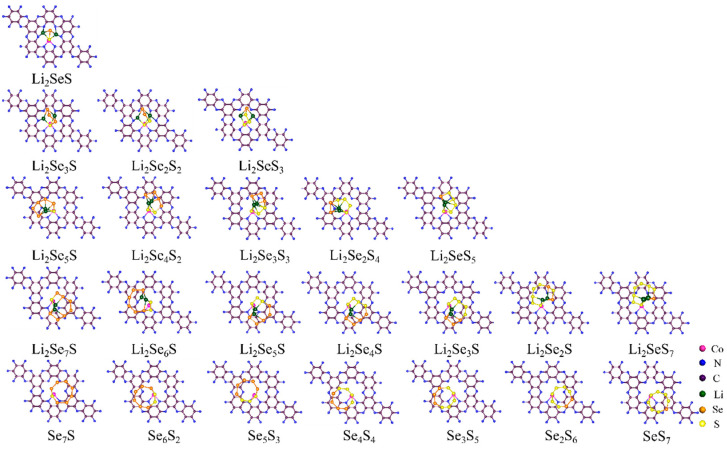
The most stable configurations of Se*_x_*S*_y_* and Li_2_Se*_x_*S*_y_* clusters anchored on the Co@C_2_N monolayer. All considered structures are shown in Table S2 of the Supporting Information.

Figure 4 shows the most stable structures of Li_2_Se*_x_*S*_y_* and Se*_x_*S*_y_* clusters adsorbed on the surface of the Co@C_2_N monolayer. Noticeably, Li_2_Se*_x_*S*_y_* (*x* + *y* = 6, 8) and Se*_x_*S*_y_* clusters align parallel to the Co@C_2_N monolayer, whereas the Li_2_Se*_x_*S*_y_* (*x* + *y* = 2, 4) clusters tend to orient perpendicular to the Co@C_2_N monolayer. This finding is similar to the situations of M@C_2_N-Li_2_S*_n_* (M = Mn, Fe, Co, Ni, Cu) [31]. As depicted in Table S3 of the Supporting Information, the average distance (*d*_S-Co@C₂N_) between S atoms and the Co@C_2_N monolayer diminishes with the decreasing concentrations of S and Se in Li_2_Se*_x_*S*_y_* clusters, which reaches the minimum distance of 2.228 Å for Co@C_2_N-Li_2_SeS. Specifically, for the Li_2_Se*_x_*S*_y_* clusters with the same composition (*x* + *y* = 4), the average distances from S atoms to the Co@C_2_N monolayer decrease during the increase in the S concentration, which is beneficial for forming strong Co-S bonds. In contrast, for the Li_2_Se*_x_*S*_y_* clusters, where *x* + *y* = 6 and 8, significant variations in the *d*_S-Co@C₂N_ values are discovered. Such differences can be attributed to the complexities arising from the increased concentration of S atoms and their varying distances to the Co@C_2_N monolayer. In addition, with the decrease in Se and S atoms, Li atoms migrate towards the Co@C_2_N monolayer, thereby decreasing the average Li-N bond length from Co@C_2_N-Li_2_Se*_x_*S*_y_* (*x* + *y* = 8) to Co@C_2_N-Li_2_SeS, aligning with the binding energies analysis. The results underscore the capability of the Co@C_2_N monolayer to enhance the anchoring and cycling stability of Li-Se*_x_*S*_y_* batteries.

### 2.3. Charge Transfer Mechanism and Electronic Properties

**Figure 5:**
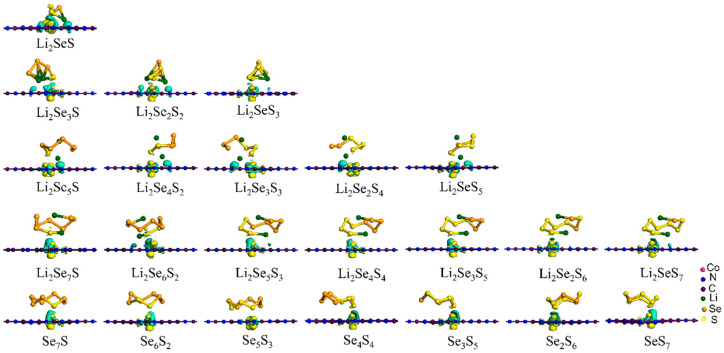
Electron density difference of Li_2_Se*_x_*S*_y_* and Se*_x_*S*_y_* clusters anchored on the surface of the Co@C_2_N monolayer. The charge density for the isovalue contour is 0.03 e Å^−3^. The cyan and yellow colors refer to the charge accumulation and depletion, respectively.

In order to evaluate the anchoring mechanism of Li_2_Se*_x_*S*_y_* and Se*_x_*S*_y_* clusters anchored on the surface of the Co@C_2_N monolayer, the charge density difference [36] and Mulliken charge analysis [37,38] are explored for the Co@C_2_N-Li_2_Se*_x_*S*_y_*/Se*_x_*S*_y_*. The charge density difference is expressed as follows:

(2) $$\Delta \rho =\rho_{Co@C_{2}N+Li_{2}Se_{x}S_{y}/Se_{x}S_{y}}-\rho_{Co@C_{2}N}-\rho_{Li_{2}Se_{x}S_{y}/Se_{x}S_{y}}$$

where $\rho_{Li_{2}Se_{x}S_{y}/Se_{x}S_{y}}$, $\rho_{Co@C_{2}N}$, and $\rho_{Co@C_{2}N+Li_{2}Se_{x}S_{y}/Se_{x}S_{y}}$ represent the charge density differences of Li_2_Se*_x_*S*_y_* and Se*_x_*S*_y_* clusters, the independent Co@C_2_N monolayer, and Li_2_Se*_x_*S*_y_* and Se*_x_*S*_y_* clusters anchored on the Co@C_2_N monolayer, respectively. As depicted by charge density difference analysis in Figure 5, significant charge transfer occurs between Li_2_Se*_x_*S*_y_*/Se*_x_*S*_y_* clusters and the Co@C_2_N monolayer, i.e., the Li and Co atoms lose electrons and the charges accumulate near the C and N atoms.

**Figure 6:**
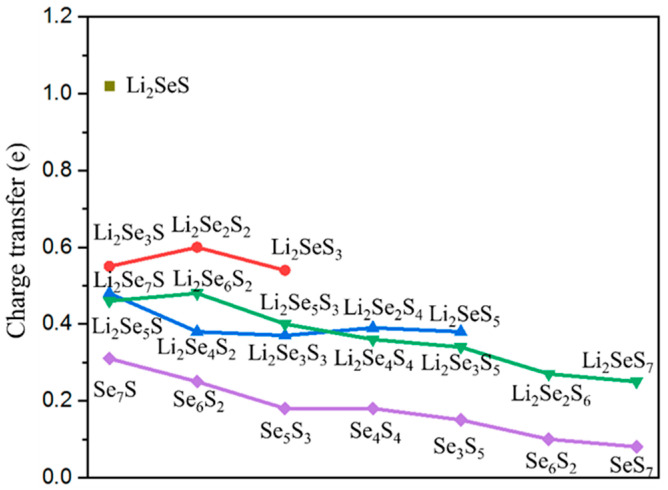
Mulliken charge transfer between Li_2_Se*_x_*S*_y_*/Se*_x_*S*_y_* clusters and the Co@C_2_N monolayer.

Notably, the charges accumulation is evident near the Li-N and Co-S bonds, whereas a reduction in the charge density is observed near the Li-S bond. These findings suggest a strengthening of the Li-N and Co-S bonds, alongside a weakening of the Li-S bonds. Furthermore, the charge densities between Li_2_Se*_x_*S*_y_* and the Co@C_2_N monolayer increase with the decrease in S and Se contents. Therefore, Li_2_Se*_x_*S*_y_* clusters exhibit robust binding to Co@C_2_N monolayer by strong Li-N and Co-S bonds, thus effectively suppressing the shuttle effect of Li_2_Se*_x_*S*_y_* and Se*_x_*S*_y_* clusters. Furthermore, the amount of charge transfer between Li_2_Se*_x_*S*_y_*/Se*_x_*S*_y_* clusters and the Co@C_2_N monolayer is analyzed through Mulliken charge calculations, as depicted in Figure 6. Obviously, positive Mulliken charges are discovered for the Li_2_Se*_x_*S*_y_*/Se*_x_*S*_y_* clusters, indicating that the charge transfers from these clusters to the Co@C_2_N monolayer. The specific charge transfer values of Co@C_2_N-Li_2_Se*_x_*S*_y_*/Se*_x_*S*_y_* clusters range from 0.31 e to 1.15 e. This electron transfer enhances the chemical interaction between Li_2_Se*_x_*S*_y_*/Se*_x_*S*_y_* clusters and the Co@C_2_N monolayer, resulting in the elongation of Li-Se and Li-S bonds in Li_2_Se*_x_*S*_y_* clusters. Notably, a positive correlation is observed between charge transfer and binding energies. For example, Co@C_2_N-Li_2_Se*_x_*S*_y_* exhibits lower charge transfer and poorer anchoring performance in comparison with the counterparts for the Co@C_2_N-Se*_x_*S*_y_*.

**Figure 7:**
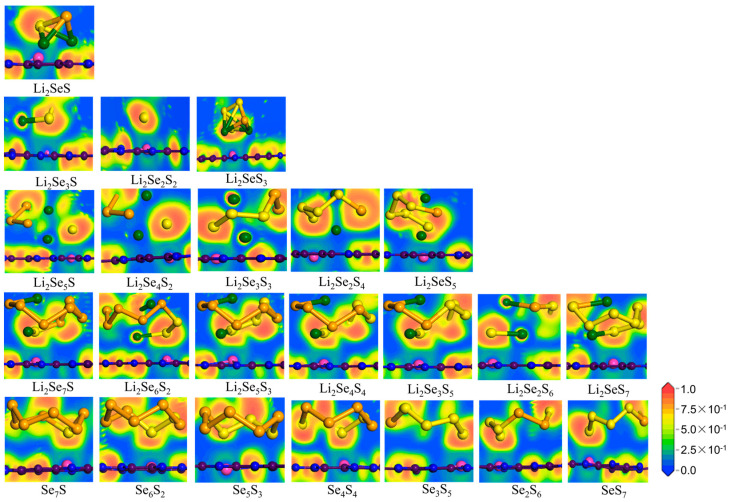
Electron localization function (ELF) plots of Li_2_Se*_x_*S*_y_*/Se*_x_*S*_y_* clusters adsorbed on the Co@C_2_N monolayer.

Figure 7 illustrates the electron localization function (ELF) [39,40] of the Li-N and Co-S bonds in Co@C_2_N-Li_2_Se*_x_*S*_y_*. In general, the ELF values are located in the range of 0 to 1, and those within 0.50~0.75 (0.75~1.00) are related to the metallic (covalent) bond. Conversely, an ELF value between 0 and 0.5 signifies an ionic bond characterized by robust bonding energy. The ELF analysis reveals the formation of stable bonds between Li_2_Se*_x_*S*_y_*/Se*_x_*S*_y_* clusters and the Co@C_2_N monolayer, i.e., the Co@C_2_N monolayer can effectively immobilize Li_2_Se*_x_*S*_y_* and Se*_x_*S*_y_* clusters through strong Co-S and Li-N bonds.

The ideal cathode material for Li-Se*_x_*S*_y_* batteries should possess excellent electrical conductivity, which is beneficial to provide additional electrons to enhance the redox kinetics of Li_2_Se*_x_*S*_y_* and Se*_x_*S*_y_* clusters during the cycle process. As depicted in Figure S1 of the Supporting Information, the density of states (DOS) of Co@C_2_N-Li_2_Se*_x_*S*_y_*/Se*_x_*S*_y_* is evaluated. Notably, some overlaps for the peaks corresponding to the Co atom and Li_2_Se*_x_*S*_y_*/Se*_x_*S*_y_* clusters are observed in the DOS, indicating strong orbital hybridization, consequently resulting in a robust Co-S bond. Upon anchoring the Li_2_Se*_x_*S*_y_*/Se*_x_*S*_y_* clusters on the surface of the Co@C_2_N monolayer, significant peaks of Li_2_Se*_x_*S*_y_* are observed near the Fermi level. Meanwhile, with the anchoring of Li_2_Se*_x_*S*_y_* and Se*_x_*S*_y_* clusters on the surface of the Co@C_2_N monolayer, the Co 3*d* orbitals hybridize with Li_2_Se*_x_*S*_y_* and Se*_x_*S*_y_* clusters orbitals near the Fermi level, and Co@C_2_N-Li_2_Se*_x_*S*_y_*/Se*_x_*S*_y_* systems exhibit metallic properties. Such finding underscores the promising prospect of Co@C_2_N monolayers as a candidate for Li-Se*_x_*S*_y_* batteries. Further analysis on the DOS near the Fermi level reveals the dominant contribution of Li_2_Se*_x_*S*_y_* clusters, whereas the corresponding contribution of the Co atom primarily concentrates in the low-energy region. With commendable electrical conductivity, the Co@C_2_N monolayer is beneficial for accelerating the reversible conversion reaction of Li_2_Se*_x_*S*_y_* clusters, thus holding great significance for expediting the electrochemical reaction of Li-Se*_x_*S*_y_* batteries.

### 2.4. Average Open Circuit Voltage of Co@C_2_N-Li_2_Se_x_S_y_

**Figure 8:**
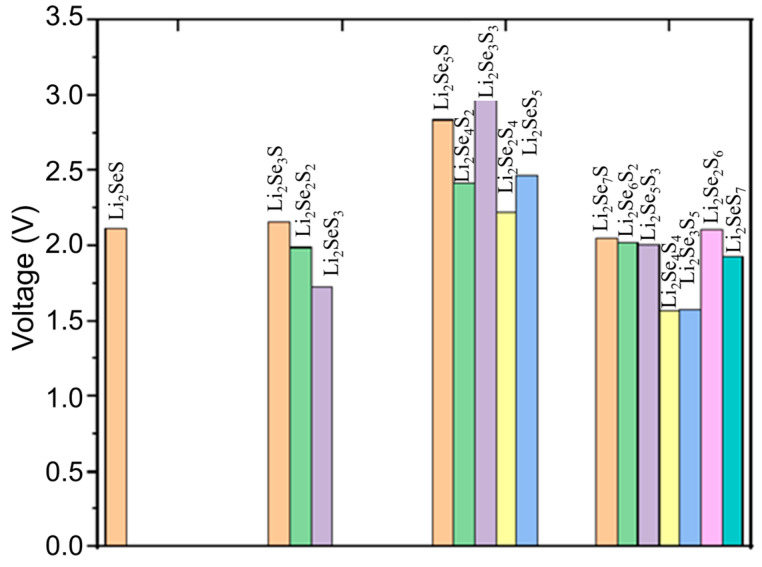
The calculated average open circuit voltages (V) of Li_2_Se*_x_*S*_y_* clusters adsorbed on the Co@C_2_N monolayer.

The average open circuit voltage (V) [41,42,43], as a key parameter for evaluating the performance of Li-Se*_x_*S*_y_* batteries, is directly estimated from the energy change during the cycling process, which is defined using the following equation:

(3) $$V=-\frac{E_{Co@C_{2}N+Li_{x_{1}}Se_{x}S_{y}}-E_{Co@C_{2}N+Li_{x_{2}}Se_{x}S_{y}}-(x_{1}-x_{2})E_{Li}}{e(x_{1}-x_{2})}$$

where $E_{Co@C_{2}N+Li_{x_{1}}Se_{x}S_{y}}$, $E_{Co@C_{2}N+Li_{x_{2}}Se_{x}S_{y}}$, and $E_{Li}$ represent the total energies of Co@C_2_N + Li*_x_*_1_Se*_x_*S*_y_*, Co@C_2_N + Li*_x_*_2_Se*_x_*S*_y_*, and metallic lithium crystals, respectively. *x*_1_ and *x*_2_ represent the number of Li atoms in the Co@C_2_N-Li*_x_1__*Se*_x_*S*_y_* and Co@C_2_N-Li*_x_2__*Se*_x_*S*_y_* structures, and e denotes the electric charge. The calculated average open circuit voltages of the Co@C_2_N-Li_2_Se*_x_*S*_y_* system are depicted in Figure 8.

Generally, the high values of average open circuit voltages are achieved through extracting one Li from the Co@C_2_N-Li_2_Se*_x_*S*_y_* systems. Notably, with the increase selenium and sulfur concentrations, the maximum average open circuit voltage of Co@C_2_N-Li_2_Se*_x_*S*_y_* (*x* + *y* = 6) reaches 2.61 V. However, with the further rising concentration of selenium and sulfur, the average open circuit voltage decreases to 1.90 V for Co@C_2_N-Li_2_Se*_x_*S*_y_* (*x* + *y* = 8).

### 2.5. Energy Profiles and the Decomposition of Li_2_SeS on the Surface of Co@C_2_N Monolayer

**Figure 9:**
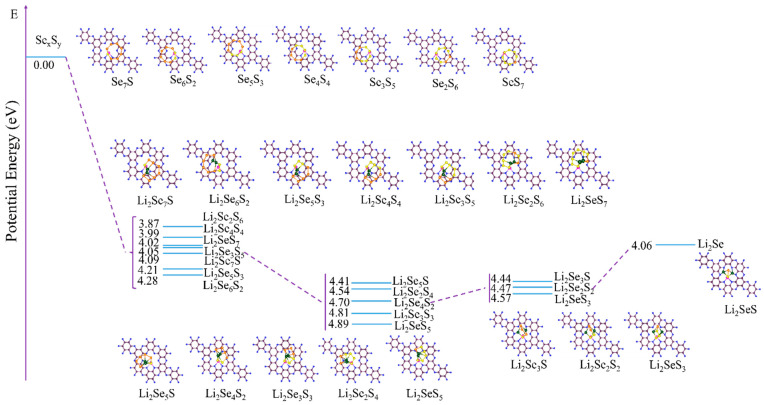
Energy profiles for the reduction of Li_2_Se*_x_*S*_y_*/Se*_x_*S*_y_* clusters on the surface of the Co@C_2_N monolayer. The magenta, blue, purple, orange, and yellow circles denote the Co, N, C, Se and S atoms, respectively.

As reported in previous studies [31,32,34,35], the transition metals deposited on the C_2_N monolayer served as the pivotal adsorption and catalytic centers, showcasing significant catalytic effect in Li-Se batteries. To unravel the reversible conversion mechanism of Li_2_Se*_x_*S*_y_* (*x* + *y* = 8) clusters on the surface of the Co@C_2_N monolayer, the energy distribution of the Se_8_ reduction pathway on the surface of the Co@C_2_N monolayer is evaluated, as depicted in Figure 9. In the discharge process, the initial step involved the reduction of two Li atoms and Se*_x_*S*_y_* to form Li_2_Se*_x_*S*_y_* (*x* + *y* = 8) clusters, followed by a sequence of reduction and disproportionation reactions, gradually yielding Li_2_Se*_x_*S*_y_* (*x* + *y* = 6), Li_2_Se*_x_*S*_y_* (*x* + *y* = 4), and Li_2_SeS clusters. Upon the spontaneous exothermic transformation from Co@C_2_N-Se*_x_*S*_y_* (*x* + *y* = 8) to Co@C_2_N-Li_2_Se*_x_*S*_y_* (*x* + *y* = 8), Co@C_2_N-Li_2_Se*_x_*S*_y_* (*x* + *y* = 6) clusters are engendered, exhibiting an average moderate exothermicity of 0.60 eV. However, in the reaction processes of Co@C_2_N-Li_2_Se*_x_*S*_y_* (*x* + *y* = 4) and Co@C_2_N-Li_2_SeS, the transformation processes become endothermic, aligning with the situations [34,36] for Li-Se batteries.

**Figure 10:**
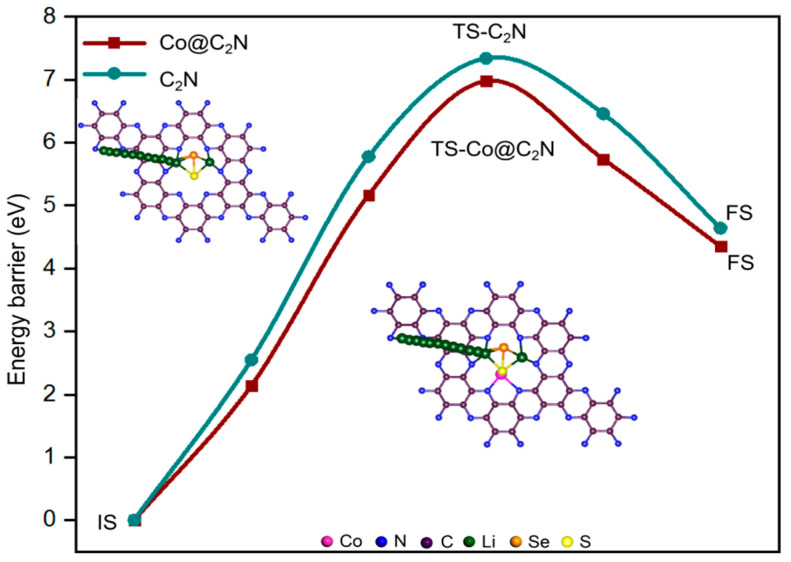
Energy barriers of the decomposition of Li_2_SeS on intrinsic C_2_N and the Co@C_2_N monolayer. The IS, TS, and FS represent the initial state, transition state, and final state along the decomposition pathway of Li_2_SeS cluster.

To elucidate the catalytic effect of Co@C_2_N monolayer on the decomposition kinetics of Li_2_Se*_x_*S*_y_* clusters, the decomposition of Li_2_SeS on Co@C_2_N and C_2_N were further evaluated as a representative example, as depicted in Figure 10. The decomposition of Li_2_SeS produces a single Li^+^ and LiSeS^−^ species by the reaction of Li_2_SeS→LiSeS^−^ + Li^+^ with an activation barrier of 7.34 eV, which is 0.36 eV lower than the counterpart observed without Co doping. Such a finding suggests that the presence of the Co atom in the Co@C_2_N monolayer reduces the decomposition energy of Li_2_SeS, which in turn leads to a higher utilization rate of active Se*_x_*S*_y_* species. Overall, the Co@C_2_N monolayer is beneficial for both facilitating the phase transition of Li_2_SeS and promoting the redox reaction.

## 3. Computational Methodology

All first-principles calculations were carried out using the Cambridge serial total energy package (CASTEP) module within the Materials studio (2019) software [44]. The exchange-correlation interactions were handled using the Perdew–Burke–Ernzerhof (PBE) functional within the framework of the generalized gradient approximation (GGA) [45,46]. However, considering the limitations of the GGA approach in accurately describing the strong exchange-correlation effects in the 3d orbitals of transition metals [35,47], a Hubbard parameter correction (U_Co_ = 4.0 eV) [48] was implemented to account for the self-interaction of electrons in Co 3d orbitals. Ultrasoft pseudopotentials [49] were employed to describe the interaction between valence electrons and ionic nuclei, and empirical corrections within the Grimme’s scheme [50] were utilized to describe the Van der Waals interactions. Structure optimization and electronic property calculations were performed using a cutoff energy of 450 eV, and a 20 Å vacuum region was used to avoid layered interactions caused by adjacent Co@C_2_N monolayers. Brillouin zone sampling employed Monkhorst-Pack grids set at 3 × 3 × 1 and 5 × 5 × 1 for structure optimization and electronic structure calculations, respectively, associating with the maximum force and energy convergence criteria of 0.05 eV/Å and 2 × 10^−5^ eV/atom, respectively.

## 4. Conclusions

In the work, the structure, anchoring mechanism, and catalytic performance of a Co@C_2_N monolayer as a substrate for a Se*_x_*S*_y_* composite cathode material were investigated with the help of first-principles calculations. The computational outcomes show that the pronounced synergistic effect of Co-S and Li-N bonds leads to increased anchoring performance for Li_2_Se*_x_*S*_y_*/Se*_x_*S*_y_* clusters, which in turn effectively mitigates the shuttle effect of high-order Li_2_Se*_x_*S*_y_* (*x* + *y* = 4, 6, 8) clusters. The charge density difference Mulliken charge analysis underscores a substantial charge transfer from the Li_2_Se*_x_*S*_y_* and Se*_x_*S*_y_* clusters to the Co@C_2_N monolayer, indicating a noticeable chemical interaction between them. Consequently, the Li-S and Li-Se bond lengths in Li_2_Se*_x_*S*_y_* clusters decrease with the anchoring process. Further density of states analysis reveals that the semi-metallic characteristics of the Co@C_2_N monolayer persist even after the adsorption of Li_2_Se*_x_*S*_y_* and Se*_x_*S*_y_* clusters, thereby facilitating the redox reaction. In addition, the catalytic performance of the Co@C_2_N monolayer as a candidate cathode material for Li-Se*_x_*S*_y_* batteries is evaluated by the decomposition of Li_2_SeS clusters, highlighting the role of the Co@C_2_N monolayer in promoting the formation and decomposition of Li_2_SeS during the discharge and charging processes. Overall, the Co@C_2_N monolayer emerges as a promising candidate material and catalyst for Li-Se*_x_*S*_y_* batteries with remarkable anchoring and catalytic performance. Our present work provides some theoretical insights into designing promising cathode materials for achieving faster, longer-lasting, and higher-energy-density Li-Se*_x_*S*_y_* batteries.

## Data Availability

The original contributions presented in the study are included in the article material; further inquiries for the raw calculation data can be directed to the corresponding author.

## Supplementary Materials

The following supporting information can be downloaded at: https://www.mdpi.com/article/10.3390/molecules29225264/s1, Table S1: Structure and total energy of Li_2_Se*_x_*S*_y_*/Se_x_S_y_ clusters; Table S2: Adsorbed structure and binding energy (*E*_b_) of Li_2_Se*_x_*S*_y_* and Se*_x_*S*_y_* (*x* + *y* = 2, 4, 6, 8) on the Co@C_2_N monolayer at different positions; Table S3: The average distances from the S atom to the Co@C_2_N monolayer and the average distances between Li atom and adjacent N atom of Co@C_2_N monolayer; Figure S1: The calculated density of states for Co@C_2_N-Li_2_Se*_x_*S*_y_* (Se*_x_*S*_y_*) structures.
