# Supplementary material for: Anchoring and Catalytic Performance of Co@C2N Monolayer for Rechargeable Li-SexSy Batteries: A First-Principles Calculations
Source: Molecules. 2024 Nov 7;29(22):5264. doi: 10.3390/molecules29225264 (PMC11596895; doi:10.3390/molecules29225264)
Supplement: Supplementary file 1 [file molecules-29-05264-s001.zip › molecules-3273682-supplementary.pdf]

Supporting Information

for

Anchoring and catalytic performance of Co@C_2N monolayer for rechargeable Li-Se_xS_y batteries: A first-principles calculations

Xiaojing Li,[a] Yingbo Zhang,*[a] Chenchen Liu,[b] Shuwei Tang[b]

[a]Department of Automotive Engineering, Hebei Petroleum University of Technology, Chengde 067000, China.

[b]College of Materials Science and Engineering, Liaoning Technical University, Fuxin, Liaoning 123000, China.

Corresponding author:

Yingbo Zhang

E-mail: zyb830327@163.com

Contents

Table S1. Structure and total energy of $Li_2Se_xS_y/Se_xS_y$ clusters…………...……….. S3

Table S2. Adsorbed structure and binding energy (E_b) of $Li_2Se_xS_y$ and Se_xS_y ($x+y$ =2, 4, 6, 8) on Co@C_2N monolayer at different positions……………………………….. S8

Table S3. The average distances from the S atom to the Co@C_2N monolayer and the average distances between Li atom and adjacent N atom of Co@C_2N monolayer…..S13

Figure S1. The calculated density of states (DOS) for Co@C_2N-$Li_2Se_xS_y/Se_xS_y$ structures……………………………………………………………………....S14

Table S1. Structure and total energy of $Li_2Se_xS_y$/Se_xS_y clusters.

$Li_2Se_xS_y$ (Se_xS_y)	Structure					
Li_2SeS	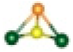<br>*E*=-4135.42 eV					
Li_2SeS_3	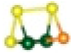<br>*E*=-4743.62eV	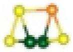<br>*E*=-4743.36 eV				
$Li_2Se_2S_2$	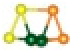<br>*E*=-7864.29 eV	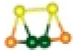<br>*E*=-7863.75 eV	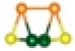<br>*E*=-7863.83 eV	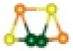<br>*E*=-7863.78 eV		
Li_2Se_3S	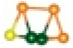<br>*E*=-10984.98 eV	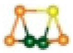<br>*E*=-10983.94 eV				
Li_2SeS_5	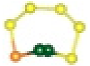<br>*E*=-5351.16 eV	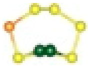<br>*E*=-5349.54eV	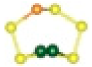<br>*E*=-5349.48 eV			
$Li_2Se_2S_4$	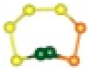<br>*E*=-8470.83 eV	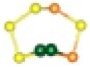<br>*E*=-8469.44 eV	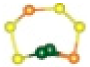<br>*E*=-8469.46 eV	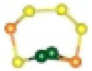<br>*E*=-8469.51 eV	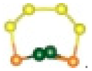<br>*E*=-8469.58 eV	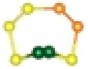<br>*E*=-8469.55 eV
	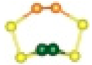<br>*E*=-8470.21 eV	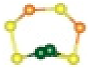<br>*E*=-8469.81 eV	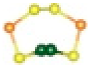<br>*E*=-8469.75 eV			

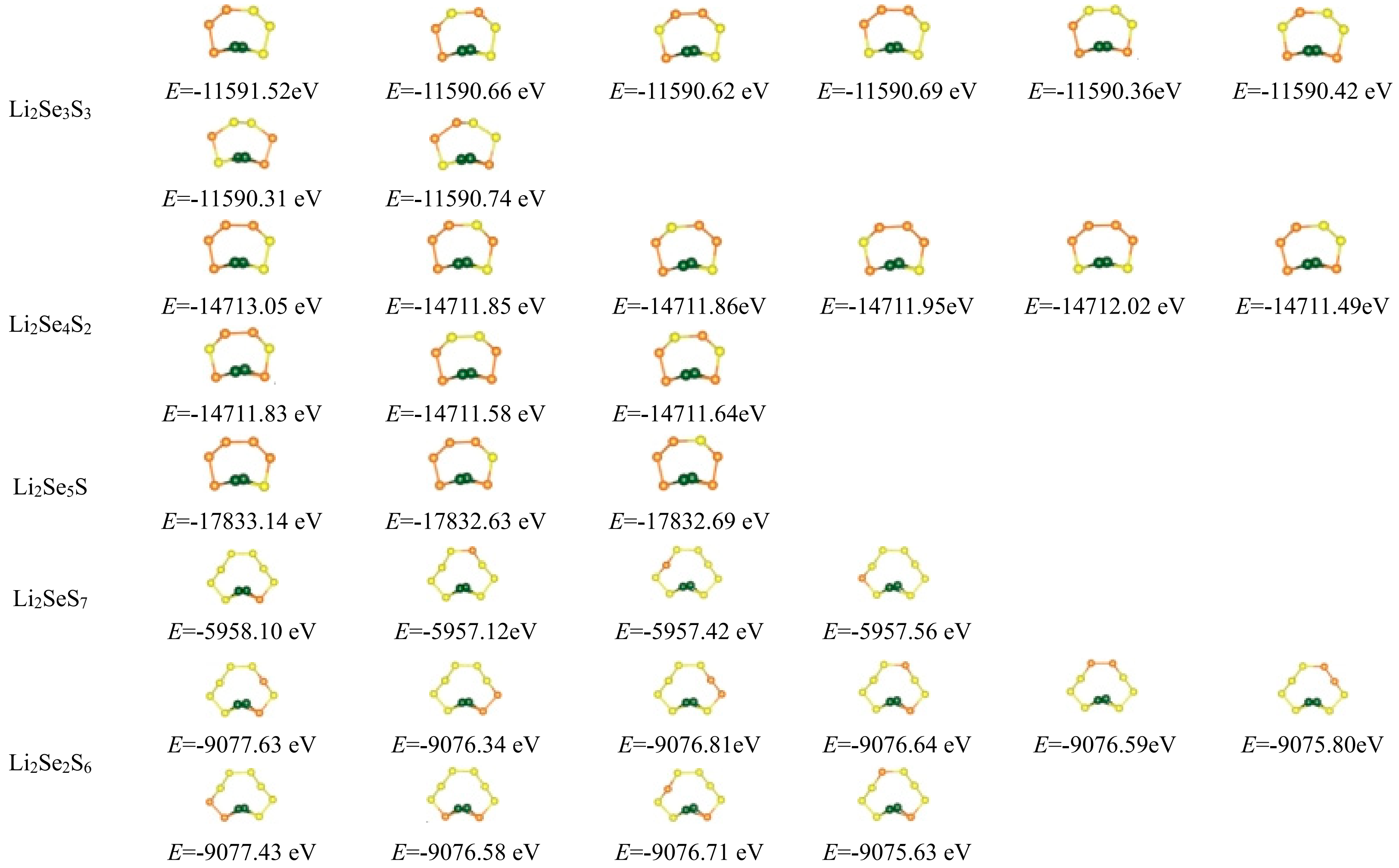
$Li_2Se_3S_3$
E=-11591.52eV
E=-11590.66 eV
E=-11590.62 eV
E=-11590.69 eV
E=-11590.36eV
E=-11590.42 eV
E=-11590.31 eV
E=-11590.74 eV
$Li_2Se_4S_2$
E=-14713.05 eV
E=-14711.85 eV
E=-14711.86eV
E=-14711.95eV
E=-14712.02 eV
E=-14711.49eV
E=-14711.83 eV
E=-14711.58 eV
E=-14711.64eV
Li_2Se_5S
E=-17833.14 eV
E=-17832.63 eV
E=-17832.69 eV
Li_2SeS_7
E=-5958.10 eV
E=-5957.12eV
E=-5957.42 eV
E=-5957.56 eV
$Li_2Se_2S_6$
E=-9077.63 eV
E=-9076.34 eV
E=-9076.81eV
E=-9076.64 eV
E=-9076.59eV
E=-9075.80eV
E=-9077.43 eV
E=-9076.58 eV
E=-9076.71 eV
E=-9075.63 eV

$Li_2Se_3S_5$

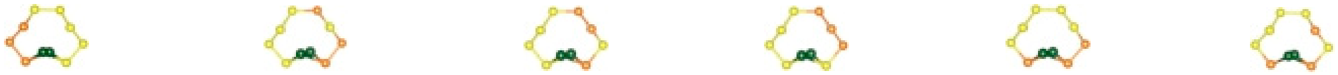

E=-12198.42 eV *E*=-12197.85 eV *E*=-12197.79 eV *E*=-12198.01 eV *E*=-12197.51 eV *E*=-12197.54 eV

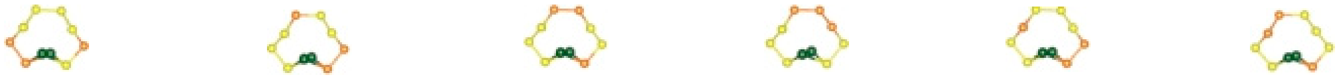

E=-12197.51 eV *E*=-12197.84 eV *E*=-12197.75eV *E*=-12197.93eV *E*=-12197.65 eV *E*=-12197.47 eV

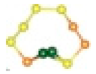

E=-12197.63eV

$Li_2Se_4S_4$

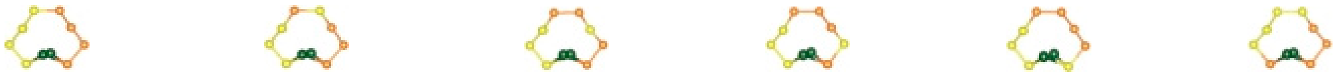

E=-15319.10 eV *E*=-15318.02 eV *E*=-15318.05 eV *E*=-15317.96 eV *E*=-15317.90 eV *E*=-15317.63 eV

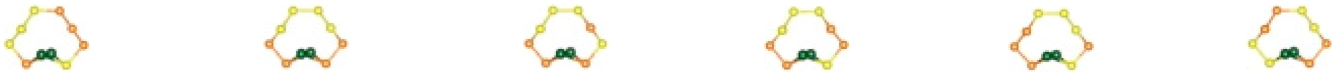

E=-15317.99 eV *E*=-15318.23 eV *E*=-15317.21eV *E*=-15317.14 eV *E*=-15317.89 eV *E*=-15317.58 eV

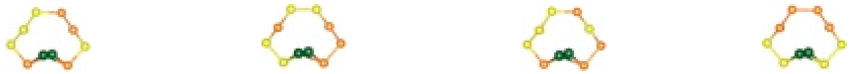

E=-15317.77 eV *E*=-15317.69 eV *E*=-15317.84 eV *E*=-15317.98 eV

$Li_2Se_5S_3$

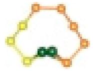
E=-18439.75 eV

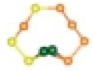
E=-18438.25 eV

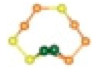
E=-18439.14 eV

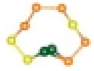
E=-18439.19 eV

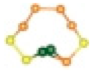
E=-18439.28 eV

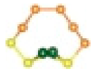
E=-18439.32 eV

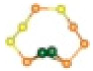
E=-18438.96eV

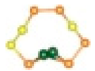
E=-18438.67 eV

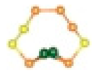
E=-18438.46 eV

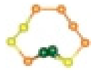
E=-18438.84 eV

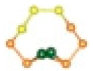
E=-18438.55 eV

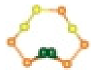
E=-184378.76 eV

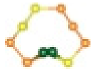
E=-18438.21eV

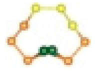
E=-18438.56 eV

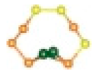
E=-18438.71 eV

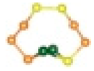
E=-18438.28eV

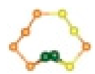
E=-18438.49 eV

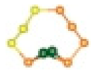
E=-18438.83 eV

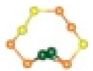
E=-18438.94 eV

$Li_2Se_6S_2$

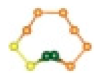
E=-21561.37 eV

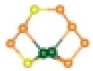
E=-21560.34 eV

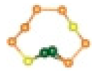
E=-21560.38 eV

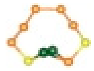
E=-21560.30 eV

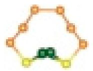
E=-21560.46 eV

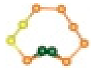
E=-21560.00 eV

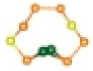
E=-21560.38 eV

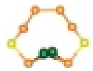
E=-21560.49 eV

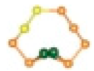
E=-21560.67 eV

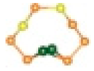
E=-21560.17 eV

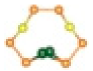
E=-21560.52eV

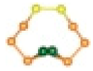
E=-21560.39 eV

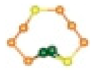
E=-21560.34 eV

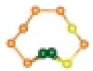
E=-21560.37 eV

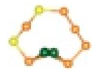
E=-21560.74 eV

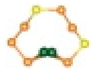
E=-21560.26 eV

Li_2Se_7S

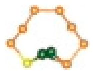
E=-24682.08 eV

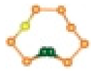
E=-24681.21 eV

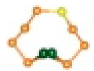
E=-24681.16 eV

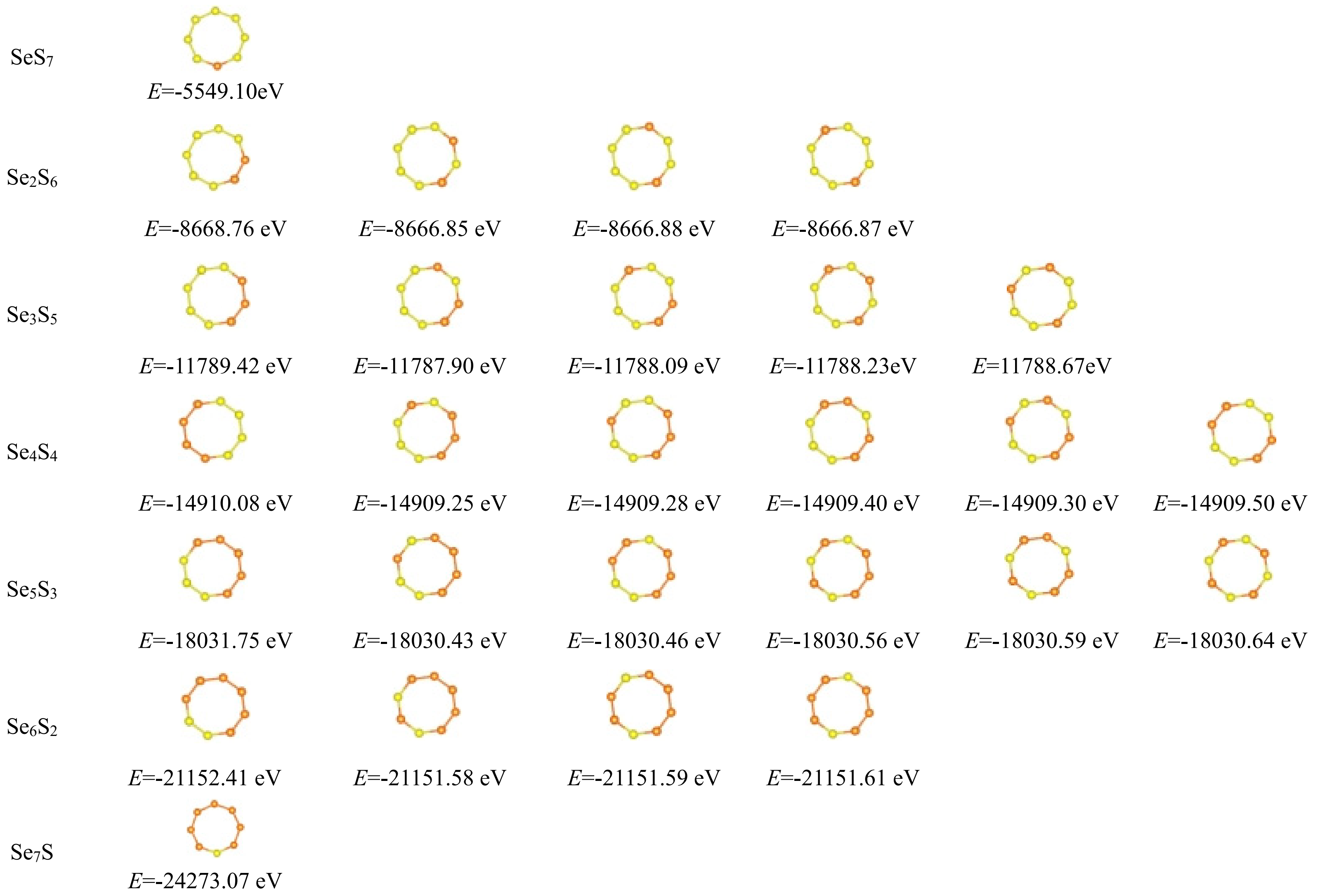
SeS7
E=-5549.10eV
Se2S6
E=-8668.76 eV
E=-8666.85 eV
E=-8666.88 eV
E=-8666.87 eV
Se3S5
E=-11789.42 eV
E=-11787.90 eV
E=-11788.09 eV
E=-11788.23eV
E=11788.67eV
Se4S4
E=-14910.08 eV
E=-14909.25 eV
E=-14909.28 eV
E=-14909.40 eV
E=-14909.30 eV
E=-14909.50 eV
Se5S3
E=-18031.75 eV
E=-18030.43 eV
E=-18030.46 eV
E=-18030.56 eV
E=-18030.59 eV
E=-18030.64 eV
Se6S2
E=-21152.41 eV
E=-21151.58 eV
E=-21151.59 eV
E=-21151.61 eV
Se7S
E=-24273.07 eV

Table S2. Adsorbed structure and binding energy (E_b) of $Li_2Se_xS_y$ and Se_xS_y ($x+y$ =2, 4, 6, 8) on Co@C_2N monolayer at different positions.

Structure / E_b				
Li_2SeS	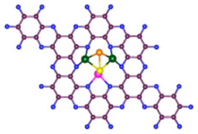	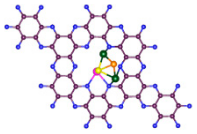	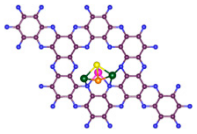	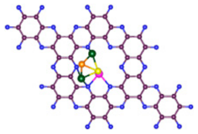
	E_b=-2.42 eV	E_b=-1.58 eV	E_b=-1.28 eV	E_b=-1.05 eV
Li_2Se_3S	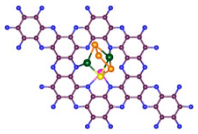	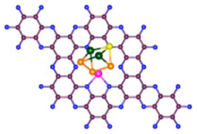	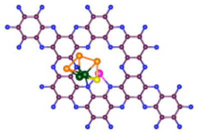	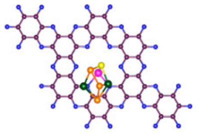
	E_b=-2.17 eV	E_b=-1.23 eV	E_b=-0.89 eV	E_b=-0.87 eV
$Li_2Se_2S_2$	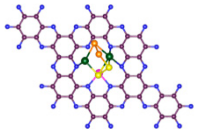	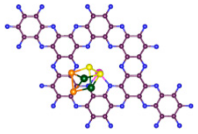	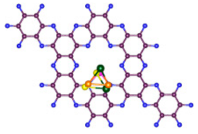	
	E_b=-2.24 eV	E_b=-1.28 eV	E_b=-1.03 eV	
Li_2SeS_3	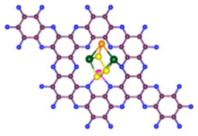	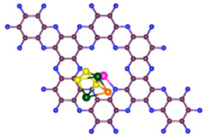	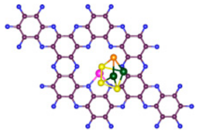	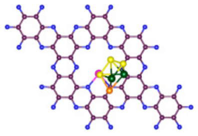
	E_b=-2.33 eV	E_b=-1.35 eV	E_b=-0.84 eV	E_b=-0.51 eV

Li_2Se_5S

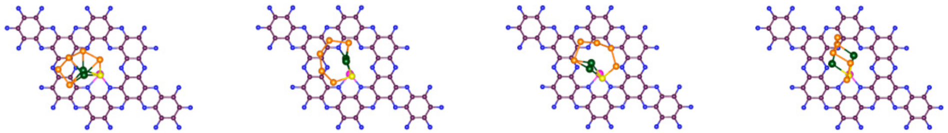

E_b=-2.02 eV E_b=-1.97 eV E_b=-1.86 eV E_b=-0.86 eV

$Li_2Se_4S_2$

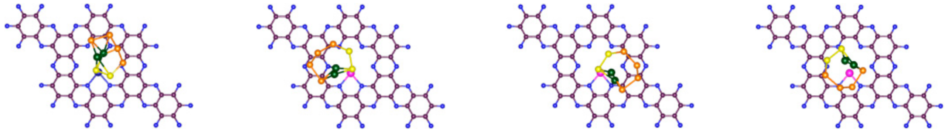

E_b=-2.24 eV E_b=-1.95 eV E_b=-1.76 eV E_b=-1.15 eV

$Li_2Se_3S_3$

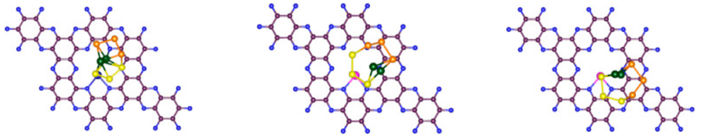

E_b=-2.23 eV E_b=-1.19 eV E_b=-0.78 eV

$Li_2Se_2S_4$

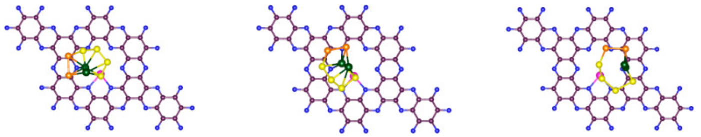

E_b=-2.24 eV E_b=-1.60 eV E_b=-0.44 eV

Li_2SeS_5

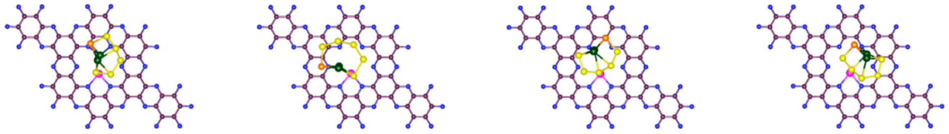

E_b=-2.33 eV E_b=-2.03 eV E_b=-1.41 eV E_b=-1.31 eV

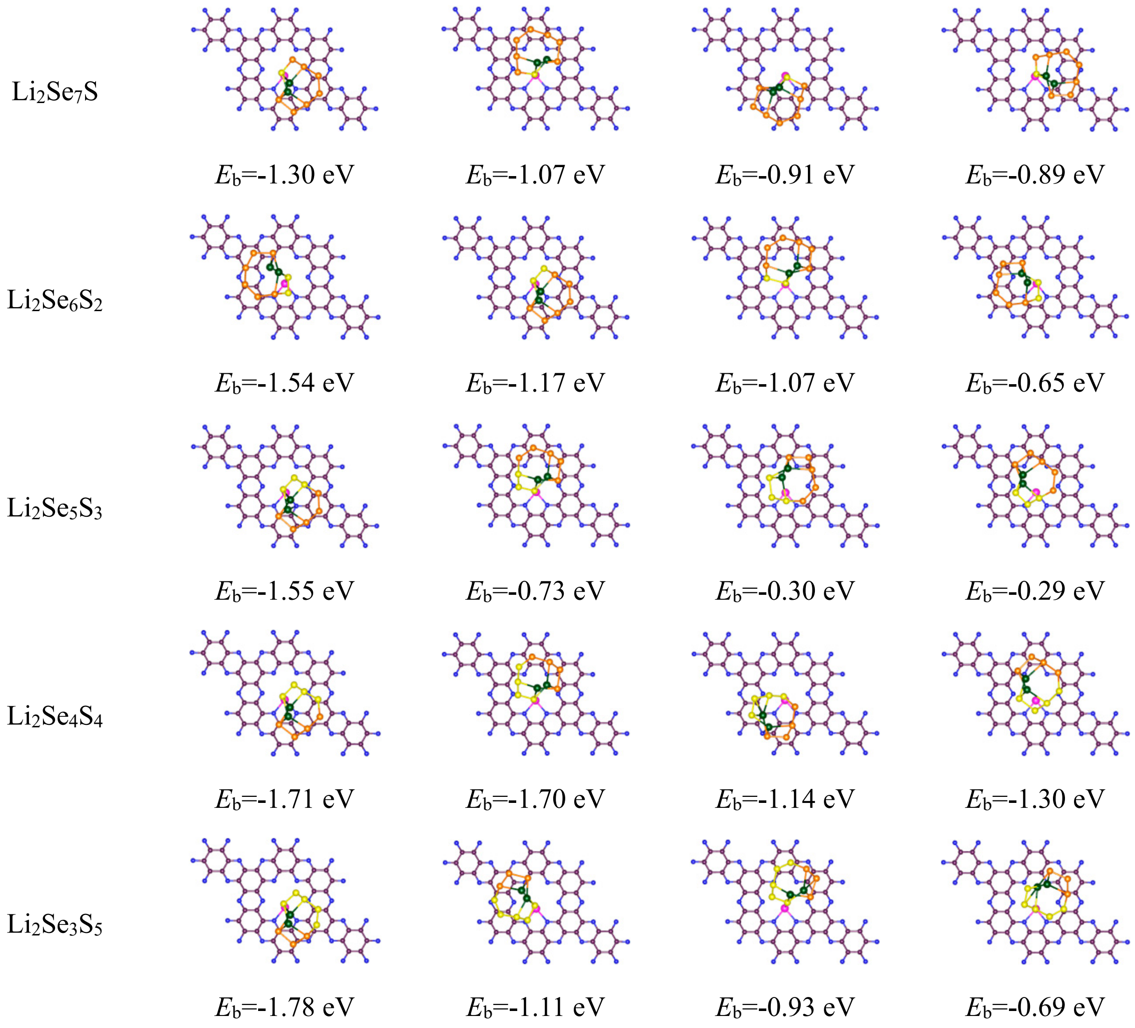
Li2Se7S
Eb=-1.30 eV
Eb=-1.07 eV
Eb=-0.91 eV
Eb=-0.89 eV
Li2Se6S2
Eb=-1.54 eV
Eb=-1.17 eV
Eb=-1.07 eV
Eb=-0.65 eV
Li2Se5S3
Eb=-1.55 eV
Eb=-0.73 eV
Eb=-0.30 eV
Eb=-0.29 eV
Li2Se4S4
Eb=-1.71 eV
Eb=-1.70 eV
Eb=-1.14 eV
Eb=-1.30 eV
Li2Se3S5
Eb=-1.78 eV
Eb=-1.11 eV
Eb=-0.93 eV
Eb=-0.69 eV

$Li_2Se_2S_6$

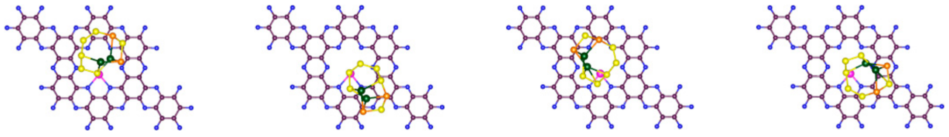

E_b=-1.75 eV E_b=-1.71 eV E_b=-1.38 eV E_b=-1.37 eV

Li_2SeS_7

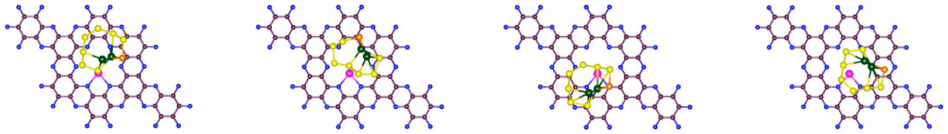

E_b=-1.75 eV E_b=-1.68 eV E_b=-1.50 eV E_b=-1.34 eV

Se_7S

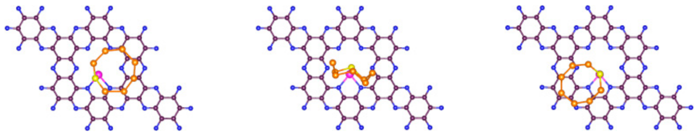

E_b=-1.11 eV E_b=-0.25 eV E_b=-0.15 eV

Se_6S_2

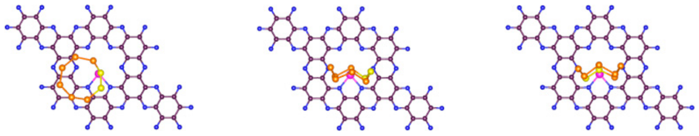

E_b=-1.10 eV E_b=-1.08 eV E_b=-0.84 eV

Se_5S_3

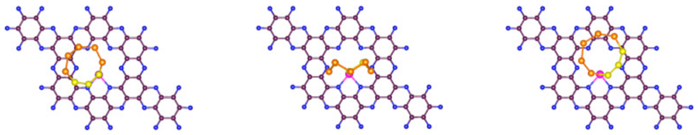

E_b=-1.13 eV E_b=-1.05 eV E_b=-0.98 eV

Se_4S_4	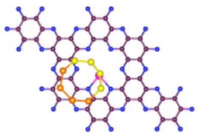	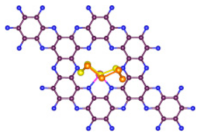	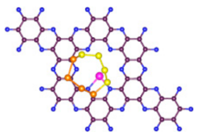	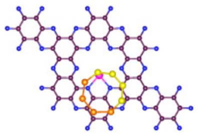
	E_b=-1.15 eV	E_b=-1.10 eV	E_b=-0.94 eV	E_b=-0.83 eV
Se_3S_5	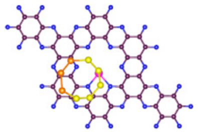	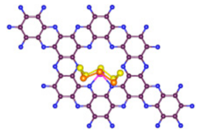	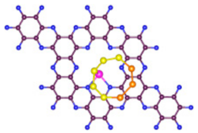	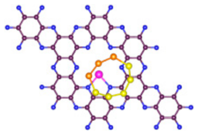
	E_b=-1.16 eV	E_b=-1.13 eV	E_b=-1.07 eV	E_b=-0.83 eV
Se_2S_6	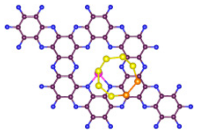	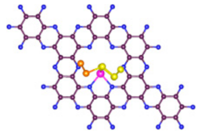	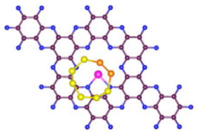	
	E_b=-1.16 eV	E_b=-1.08 eV	E_b=-0.91 eV	
SeS_7	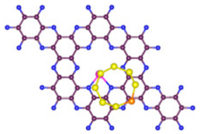	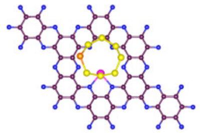	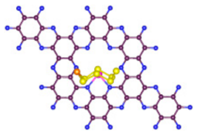	
	E_b=-1.15 eV	E_b=-1.08 eV	E_b=-1.07 eV	

Table S3. The average distances from the S atom to the Co@C2N monolayer ($d_{S\text{-}Co@C_2N}$, Å), and the average distances between Li atom and adjacent N atom of Co@C2N ($d_{Li\text{-}N}$, Å).

**Species**		$d_{S\text{-}Co@C_2N}$**(Å)**	$d_{Li\text{-}N}$**(Å)**
Li_2SeS	Li_2SeS	2.228	2.136
$Li_2Se_xS_y$ (x+y=4)	Li_2Se_3S	2.390	2.538
	$Li_2Se_2S_2$	2.386	2.432
	Li_2SeS_3	2.331	2.316
$Li_2Se_xS_y$ (x+y=6)	Li_2Se_5S	2.400	2.620
	$Li_2Se_4S_2$	2.360	2.665
	$Li_2Se_3S_3$	2.411	2.639
	$Li_2Se_2S_4$	2.396	2.632
	Li_2SeS_5	2.387	2.619
$Li_2Se_xS_y$ (x+y=8)	Li_2Se_7S	2.504	2.989
	$Li_2Se_6S_2$	2.573	2.704
	$Li_2Se_5S_3$	2.515	2.940
	$Li_2Se_4S_4$	2.441	2.904
	$Li_2Se_3S_5$	2.418	2.908
	$Li_2Se_2S_6$	2.415	2.905
	Li_2SeS_7	2.417	2.708
Se_xS_y (x+y=8)	Se_7S	2.359	
	Se_6S_2	2.479	
	Se_5S_3	2.470	
	Se_4S_4	2.456	
	Se_3S_5	2.456	
	Se_2S_6	2.478	
	SeS_7	2.410	

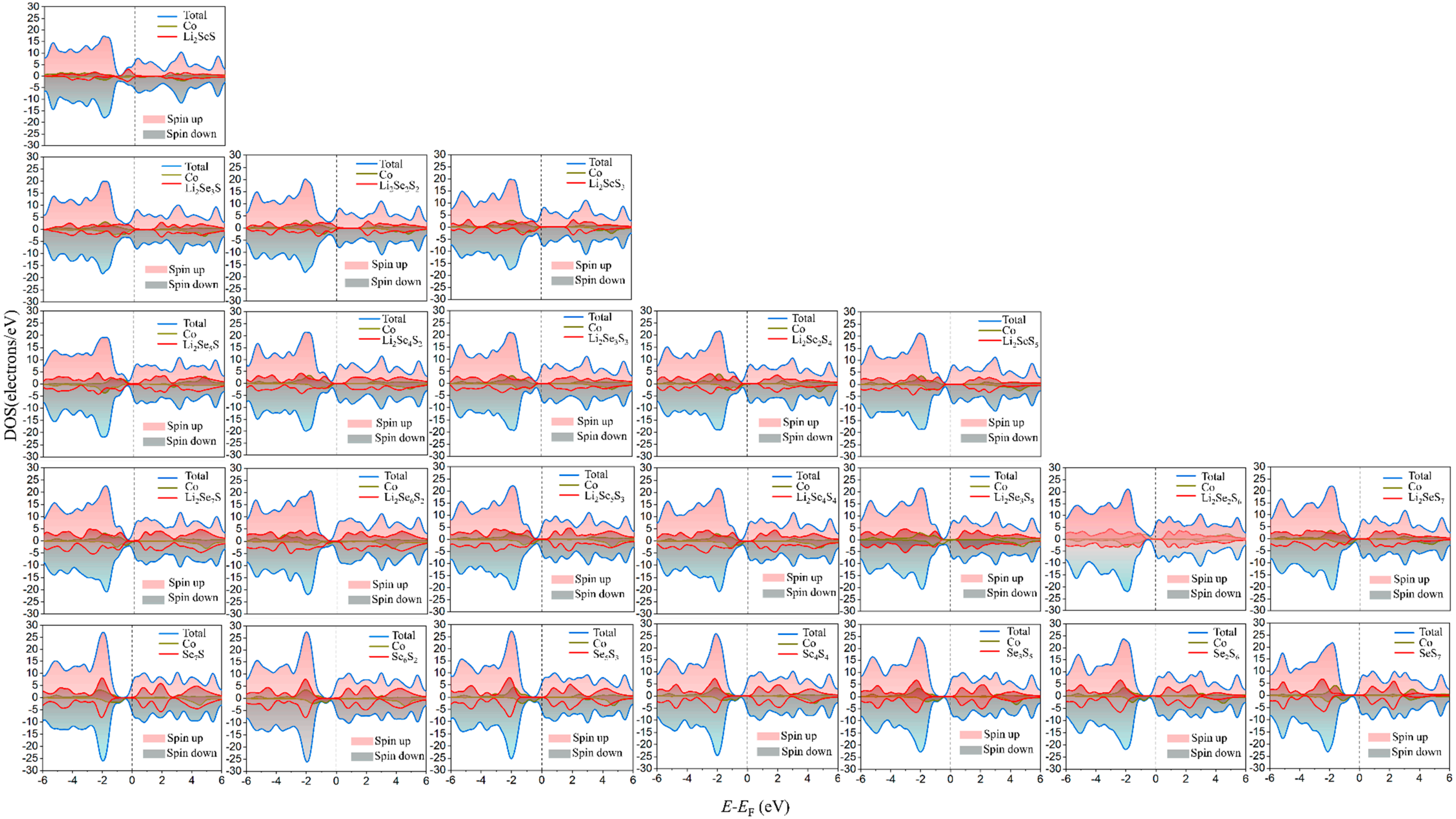

Figure S1. The calculated density of states (DOS) for Co@C2N-Li2SexSy/SexSy structures. The dashed lines represent the Fermi level.
